# Can Accretion Products Be Formed at the Interface of Freshly Nucleated Particles?

**DOI:** 10.1021/acsomega.5c13316

**Published:** 2026-03-30

**Authors:** Galib Hasan, Theo Kurtén, Ivo Neefjes, Jonas Elm

**Affiliations:** † Department of Chemistry, 1006 Aarhus University, Langelandsgade 140, Aarhus 8000, Denmark; ‡ Department of Chemistry, 3835 University of Helsinki, P.O. Box 55 (A.I. Virtanens plats 1), Helsinki FIN-00014, Finland

## Abstract

Oligomerization reactions from RO_2_ + R′O_2_ radicals, occurring via a triplet (RO···^3^O_2_···OR′) cluster, are an important gas-phase reaction for the formation of low-volatile ROOR′ accretion products. However, it remains unknown whether such reactions can occur at the interface of freshly nucleated particles (FNPs). For instance, FNPs coated with a shell of organic compounds could potentially form accretion products at the surface, further stabilizing the particle. Using quantum chemical methods, we here study how the RO_2_ + R′O_2_ reaction is influenced by interaction with FNP precursors such as sulfuric acid (SA), ammonia (AM), and dimethylamine (DMA). For the RO_2_′s, we tested simple branched hydroxyl peroxy radicals (HO–RO_2_) as the tether to the FNP components. Cluster structures were obtained using a systematic conformational sampling approach based on the ABCluster program and CREST. We calculated the final structure and vibrational frequencies at the ωB97X-D/6–31++G­(d,p) level of theory. Energy levels for intersystem crossing calculations were carried out at the XMC-QDPT2/6–311++G­(d,p) level of theory, and spin–orbit coupling matrix elements were calculated using CASSCF­(6,4)/6–311++G­(d,p). Our calculations show that the rate of the intersystem crossing needed to form ROOR′ accretion products at the FNP model clusters lies in the range of 10^6^–10^9^ s^–1^, similar to the rate previously computed in the gas phase. We also find that both the intermediate (RO···^3^O_2_···OR′) clusters and the resulting ROOR′ accretion products interact strongly with the FNP components, leading to suppressed evaporation *if* formed at the surface. Unfortunately, the formation is limited by the requirement of two RO_2_ radicals being involved. We hypothesize a pathway where the RO_2_/R′O_2_ are formed via oxidation reactions at the surface and recombine via a Langmuir–Hinshelwood mechanism. However, this must still be considered an extremely rare event.

## Graphical abstract

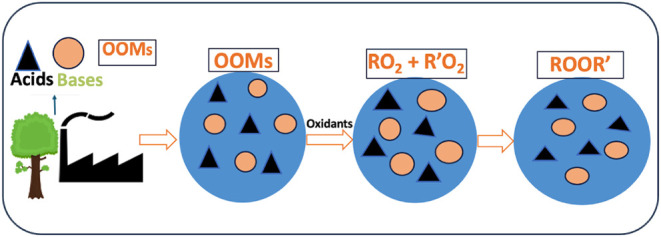

## 1 Introduction

Aerosol particles are omnipresent in the ambient atmosphere. Fine particles (<1 μm in diameter) and ultrafine particles (<100 nm in diameter), are responsible for most air pollution-related mortality. Inhalation of these particles poses significant risks to public health, by increasing the likelihood of lung and cardiovascular diseases.^1,2^ Many atmospheric aerosols, particularly scattering species such as sulfate and secondary organic aerosols, contribute to a net cooling effect by reflecting incoming solar radiation. In contrast, strongly absorbing aerosols such as black carbon can exert a warming effect.^3^ Moreover, aerosol particles exert a significant indirect impact on the global climate by acting as cloud condensation nuclei (CCN), facilitating the formation of clouds, fog, and mist.^4^ To date, the interactions between aerosol particles and clouds remain the least understood process in global climate estimation.^5^ In particular, there is a significant lack of understanding of how small particles grow to larger sizes. Hence, it remains unknown how particles smaller than 3 nm behave during the early stages of growth that enable them to act as CCN.^6^

The dominant source of aerosol particles (accounting for 50–90% by number) arises from the nucleation of vapors in the atmosphere, resulting in a rapid formation of freshly nucleated particles (FNPs) that are typically 1–2 nm in size.^7^ The formation of new particles in the atmosphere is initiated by the formation of strong noncovalently hydrogen-bonded molecular clusters.^8^ Clusters that possess strong intermolecular interactions can remain stable against evaporation, allowing them to grow further into aerosol particles of 2–3 nm and above. Inorganic acids, such as sulfuric acid (SA), and bases, such as ammonia (AM) and amines like dimethyl and trimethyl amine (DMA, TMA), are key components in the initial cluster formation in the atmosphere.^9^ Additionally, ions from galactic cosmic rays and other chemical species, such as highly oxygenated organic molecules (HOMs) formed through oxidation reactions, are also thought to play a role in influencing clustering.^10,11^ Most oxygenated organic molecules (OOMs) will not be involved in the FNP formation, but rather aid in the growth after the initial inorganic core is formed. This will lead to a core–shell structure with a coat of OOMs at the surface.^12^

Oligomerization reactions of organic peroxy RO_2_ radicals, which are key intermediates in atmospheric chemistry, produce low-volatile covalently bound ROOR′ accretion products. Accretion products have been detected in both atmospheric aerosols^13,14^ and the gas phase.^15^ Due to their extremely low volatility, accretion products play a crucial role in new particle formation^16,17^ possibly even serving as nuclei for more volatile organic vapors to condense and grow upon. For example, accretion product formation prevents the constituent monomers from evaporating and enhances the stability of FNPs.

In atmospheric chemistry, chemical reactions are generally classified as either gas-phase reactions or reactions occurring within the bulk particle phase. In contrast, the surface chemistry of aerosol particles is far less understood, even though the particle surface provides a chemically distinct environment compared to the bulk. Notably, small particles have been observed to exhibit unexpected acidity, which can have significant implications for surface chemical processes.^18^ Bimolecular reactions occurring on the surface of FNPs might be catalyzed by the close proximity of acids, bases, and water molecules, which can be readily available within the FNP environment.^19^ Consequently, FNPs offer a unique environment for surface reactions, as their small diameters result in a significantly higher surface-area-to-mass ratio compared to larger particles.

We previously investigated accretion product formation from the RO_2_ + R′O_2_ reaction in the gas phase, a complex process with multiple competing channels.^20–23^ The recombination of two peroxy RO_2_ radicals proceeds through the formation of a singlet RO_4_R′ tetroxide intermediate. This tetroxide subsequently undergoes bond cleavages that yield a singlet (RO···O_2_···R′O) three-body complex, where the O_2_ molecule is in its triplet ground state. To conserve total spin, therefore, the two RO radicals within the cage must have parallel spins, resulting in a overall cluster multiplicity as triplet. The ^3^(RO···OR′) cluster, which is formed after loss of O_2_ from the three-body complex, can participate in four major reaction pathways:

1 A hydrogen shift reaction forming R_–H_ = O + R′OH.
2 A “spin-flip” (intersystem crossing) allowing the formation of ROOR′ accretion products.
3 A β-scission, allowing, if followed by an intersystem crossing, the formation of ROR′ ester/ether dimer.
4 Evaporation or dissociation into two alkoxy radicals RO + R′O.

The actual situation is more complex, as ISCs and β-scission may also be followed by H-shifts (channel 1) or dissociation (channel 4). Using state-of-the-art quantum chemical methods, we found that these channels are all potentially rapid, with typical reaction rates ranging from 10^6^–10^10^ s^–1^ strongly depending on the chemical system.^20–23^ However, the branching between these channels remains ambiguous. Experimental work suggests that when the peroxy radical is large and multifunctional, the yield of accretion product formation increases to nearly unity.^24^ This can be rationalized by the finding that as the R and R′ become more complex and functionalized, the binding energy of the ^3^(RO···OR′) clusters increases, while the H-shift rate and dissociation rate decrease.

In this study, we shifted our focus to RO_2_ + R′O_2_ reactions on FNP surface, where little is known about surface chemistry. We used a simple dimer cluster consisting of sulfuric acid (SA) and ammonia (AM)/dimethylamine (DMA) as our model FNP. We included simple alkoxy radicals (ethyl, isopropyl, acetonyl, and 2-oxyl-1-butanol), with an alcohol group (OH) attached to one carbon and an alkoxy oxygen at the other end carbon, abbreviated as HO-EtO, HO-iPrO, AcO and BuOH-O, respectively. Many of these systems correspond to those experimentally studied by Berndt et al.^25^ but we added an additional OH group as the “anchor” between the reactants and the FNP components. These systems remain atmospherically relevant, for instance, Murphy et al.^26^ recently studied gas-phase accretion product formation from OH-peroxy radicals.

In this study we will explore three possible mechanisms by which ROOR′ accretion products can attach to the FNP surface. Finally, we combine quantum chemical simulation of FNP–organic cluster structures with binding free energy analyses to assess the feasibility of RO_2_ + R′O_2_ accretion product formation when interacting with FNP components. By comparing binding energies, evaporation lifetimes, and collision-driven kinetics, we evaluate whether FNPs can realistically act as platforms for accretion product chemistry in the atmosphere.

## 2 Methodology

### 2.1 Computational Details

Density functional theory (DFT) calculations during the configurational sampling procedures (single point, geometry optimization and vibrational frequency calculations) were performed at the ωB97X-D/6–31++G­(d,p) level of theory using Gaussian16 Rev. B.01. GFN1-xTB^27^ semiempirical calculations were performed using the xTB 6.4.0 program.^28^ Meta-dynamics calculations were conducted using CREST in noncovalent interaction mode.^29–33^ Initial clusters were generated with ABCluster version 3.2 ^34,35^ with a CHARMM force field.^36^

For the accretion product formation, intersystem crossing (ISC) rate calculation, the energies of the four lowest singlet (*S*_1_, *S*_2_, *S*_3_, *S*_4_) and ground triplet (*T*_1_) states were computed at the XMC-QDPT2/6–311++G­(d,p) level of theory using Firefly, version 8.2.0. The spin–orbit coupling matrix elements between ground triplet *T*_1_ and singlet states *S*_1_–*S*_4_ were calculated at the CASSCF­(6,4)/6–311++G­(d,p) level of theory, while using XMC-QDPT2/6–311++G­(d,p) energies, with the general atomic and molecular electronic structure system (GAMESS-US) program.^37^

### 2.2 Studied Systems

We studied simple alkoxy radicals (ethyl, isopropyl, acetonyl, and 2-oxyl-1-butanol), with an alcohol group (OH) attached to one carbon and an alkoxy oxygen at the other end carbon. We will denote these radicals as HO–EtO, HO–iPrO, AcO and BuOH–O, respectively. We note that the OH-group is simply chosen as a tether to interact with the FNP components. Hence, the aim is not to find an organic that bind strongly to the FNP, but investigate the change in kinetics and binding affinity upon accretion product formation compared to the gas phase.

We used an (SA)_1_(base)_1_ dimer cluster, where the base is either an AM or DMA molecule, to model the FNP. While this is a simplified model, it still offers valuable insight into how the proximity of FNP components can influence accretion product formation. Hence, in this work, we employ (SA)_1_(AM)_1_ and (SA)_1_(DMA)_1_ dimer clusters as simplified molecular models representing the earliest acid–base building blocks involved in particle formation, rather than fully developed particles with extended surfaces. The aim is to extend the work to larger systems in the future by including more FNP components. For a discussion on uncertainty introduced by using a simplified model, we refer to Section S1 in the Supporting Information.

### 2.3 Configurational Sampling Workflow

Systematic configurational sampling was performed on the ^3^(RO···OR′)_1_(SA)_1_(AM)_1_ and ^3^(RO···OR′)_1_(SA)_1_(DMA)_1_ clusters, generating numerous local minima cluster conformers. The configurational sampling involved several steps employing well tested sampling protocols.^38,39^ Initially, configurations were generated using the artificial bee colony (ABC) algorithm, employing rigid-body molecules. Specifically, 10,000 local minima configuration structures were generated through 10 parallel ABCluster runs. Building on our recent studies^7,40^ we conducted multiple parallel ABCluster explorations, as they yield more accurate predictions for the global energy minimum configuration of large clusters compared to a single extended exploration. The ABC algorithm requires monomer structures, along with Lennard-Jones parameters and partial charges for all atoms within the monomers. The structures and partial charges were obtained from optimization and natural bonding orbital (NBO) calculations at the ωB97X-D/6–31++G­(d,p) level of theory, while the Lennard-Jones parameters were obtained from the CHARMM force field database.

Semiempirical optimizations were then carried out using the XTB program^27^ and the GFN1-xTB (Geometry, Frequency, Noncovalent, eXtended Tight Binding) level of theory^27^ for the 10,000 configurations generated with ABCluster. DFT single-point calculations were subsequently performed using the ωB97X-D/6–31++G­(d,p) level of theory on the conformers optimized with GFN1-xTB. Next, high-energy configurations were filtered out by selecting only the 1,000 lowest-energy structures (energy range ∼0–7.4 kcal mol^–1^) for full optimization at the ωB97X-D/6–31++G­(d,p) level. Among these, the 100 lowest-energy configurations (energy range ∼0–2.8 kcal mol^–1^) were further selected for optimization and vibrational frequency calculations. The configurational sampling process can be summarized as follows:

$$\mathrm{ABC}\overset{N=10,000}{\to }{\mathrm{xTB}}^{\mathrm{OPT}}\overset{N=10,000}{\to }\omega {\mathrm{B97X‐D}}^{\mathrm{SP}}\overset{N=1,000}{\underset{\mathrm{filter}}{\to }}\omega {\mathrm{B97X‐D}}^{\mathrm{OPT}}\overset{N=100}{\underset{\mathrm{filter}}{\to }}\omega {\mathrm{B97X‐D}}^{\mathrm{Freq}},\left(\mathrm{ABC}\;\mathrm{track}\right)$$

The identified lowest free energy conformer was then selected for CREST exploration as suggested by Knattrup et al.^41^ which involves metadynamics to provide additional configurational sampling using the following workflow:

$$\mathrm{CREST}\overset{N=100}{\to }\omega B97{\mathrm{X‐D}}^{\mathrm{Full}\;\mathrm{Opt}},\left(\mathrm{CREST}\;\mathrm{track}\right)$$

The CREST exploration was conducted using GFN1-xTB in noncovalent interaction mode. From the CREST optimization, we selected the 100 lowest-energy geometries for full optimization and vibrational frequency calculation to obtain the corresponding Gibbs free and electronic energies. The overall workflow applied here is extremely exhaustive and most likely overkill for the small clusters studied here.^39^ However, the workflow is directly extendable to more realistic sized FNPs, which will be studied in the future.

### 2.4 ISC Rate Calculation

The ^3^(RO···OR′) clusters can undergo intersystem crossing (ISC) to the singlet surface when interacting with FNP components, facilitating rapid recombination into covalently bound ROOR′ accretion products. The methodology for our ISC rate calculations has been explained in detail in our previous work.^20,21^ Briefly, we selected the global minimum configurations identified through the sampling workflow described above. The *k*_ISC_ calculation requires the spin–orbit coupling interaction matrix elements (SOCME) and the excitation energies (*E*) of the involved electronic states. The energies of the four lowest singlet and triplet states were then computed at the XMC-QDPT2/6–311++G** level of theory. The ISC rate coefficient, *k*_ISC_ (in s^–1^), was subsequently determined using the following equation:^20^

1 $$k_{\mathrm{ISC}}=1.6\times 10^{9}\left\langle i\right|{Ĥ}_{\mathrm{SO}}|f\rangle^{2\;}\mathrm{FC}$$

The term ⟨*i*|*Ĥ*_SO_|*f*⟩^2^ represents the spin–orbit coupling matrix element in cm^–1^, while FC denotes the Franck–Condon factor, which depends on the energy gap between the electronic states. To determine the most appropriate active space, we explored and tested different configurations to assess which sets of orbitals contribute significantly to state averaging. Our test revealed that the (6,4) active space—consisting of six electrons distributed across four orbitals—offers a suitable balance between computational efficiency and accuracy, making it sufficient to describe the states of interest. A detailed discussion on active space selection can be found in our previous work.^20^ The CAS­(6,4) active space includes the two singly occupied 2p orbitals localized on the radical oxygen atoms and the corresponding bonding/antibonding combinations relevant for O–O bond formation. Orbital analysis shows that, even in the presence of the sulfuric acid–base cluster, the *T*_1_ and low-lying singlet states remain dominated by these radical-centered orbitals, with negligible participation from cluster-localized orbitals. Thus, the active space required to describe the ISC mechanism is unchanged relative to the gas phase. Based on the applied level of theory, we estimate the uncertainty in the ISC rate constants calculated via eq 1) to be within approximately one to 2 orders of magnitude.^42^

## 3 Results and Discussion

### 3.1 Mechanistic Pathways of ROOR′ Formation on FNPs

The formation of the ^3^(RO···OR′)­(FNP) intermediate cluster is a necessary precondition for the formation of ROOR′ accretion products on FNPs. To evaluate the feasibility of ^3^(RO···OR′) formation on FNPs, we examined three possible mechanistic pathways (Scheme 1). For each mechanism we outline how plausible they are from a kinetic perspective of the lifetime of the species, as well as the collision rates.

**Scheme 1:**
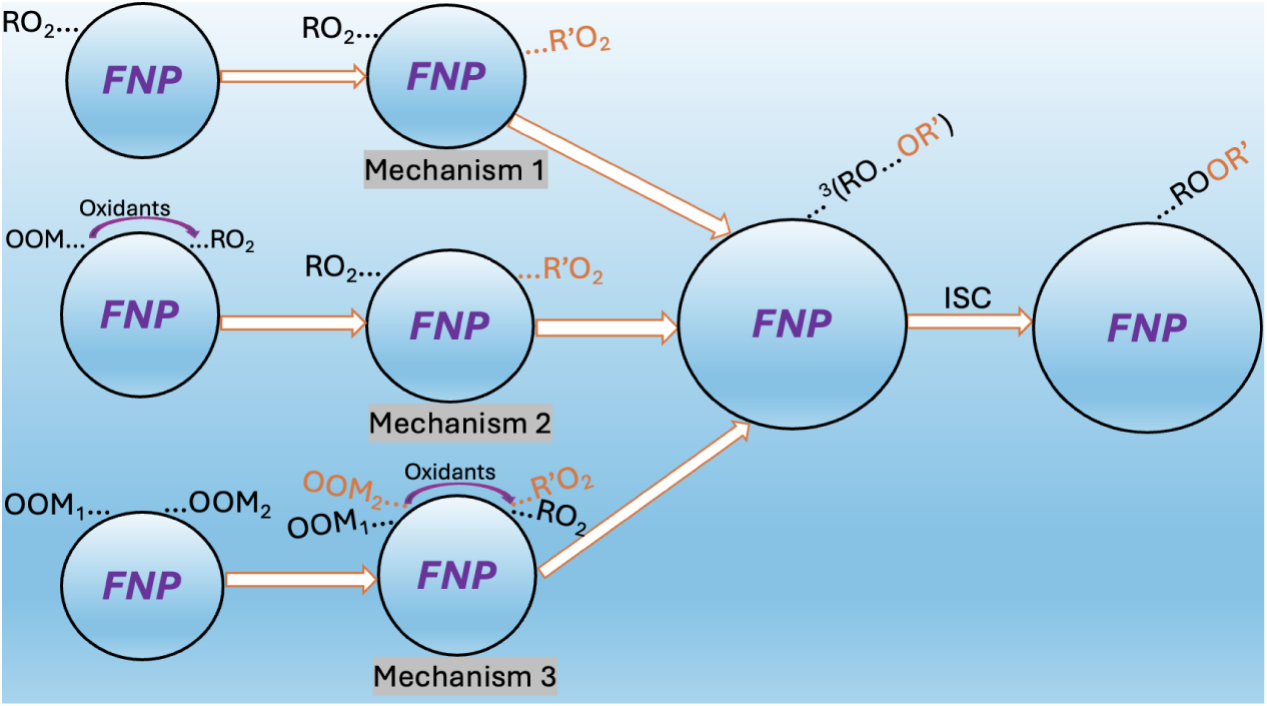
Proposed Mechanistic Pathways for the Formation of ROOR′ on Freshly Nucleated Particles (FNPs)^a^

a The key intermediate is the triplet ^3^(RO···OR′)­(FNP) cluster before ROOR′ accretion, which may form through different surface-mediated routes (see the text for details).

Mechanisms 1 and 2 follow an Eley–Rideal (ER) mechanism, where one adsorbed reactant reacts directly with an RO_2_ colliding from the gas phase. Mechanism 3 follow a Langmuir–Hinshelwood mechanism, where two RO_2_ are first adsorbed, and subsequently undergoes reactions.

#### 3.1.1 Mechanism 1: Sequential RO_2_ Addition

This pathway involves the consecutive collision of two RO_2_ radicals from the gas phase with the same FNP:

2 $${\mathrm{RO}}_{2}+\mathrm{FNP}\to \left({\mathrm{RO}}_{2}\right)\left(\mathrm{FNP}\right)$$

3 (RO2)(FNP)+R′O2→(RO···OR′)3(FNP)+O2→(ROOR′)(FNP)

#### 3.1.2 Mechanism 2: OOM Oxidation on FNP

In this case, an OOM first binds to an FNP, undergoes oxidation to (RO_2_)­(FNP), and then subsequently reacts with another RO_2_:

4 OOM+FNP→(OOM)(FNP)

5 $$\left(\mathrm{OOM}\right)\left(\mathrm{FNP}\right)+\mathrm{Oxidant}\to \left({\mathrm{RO}}_{2}\right)\left(\mathrm{FNP}\right)$$

6 (RO2)(FNP)+R′O2→(RO···OR′)3(FNP)+O2→(ROOR′)(FNP)

#### 3.1.3 Mechanism 3: Double OOM Oxidation on FNP

After FNP formation, oxygenated organic molecules (OOMs) will drive the growth of the particle. This implies that OOMs will stick to the surface and are subsequently prone to oxidant attack. Hence, two coadsorbed OOMs on an FNP surface could subsequently be oxidized to RO_2_ radicals, which combine on the surface:

7 $${\mathrm{OOM}}_{1}+{\mathrm{OOM}}_{2}+\mathrm{FNP}\to \left({\mathrm{OOM}}_{1}\right)\left({\mathrm{OOM}}_{2}\right)\left(\mathrm{FNP}\right)$$

8 $$\left({\mathrm{OOM}}_{1}\right)\left({\mathrm{OOM}}_{2}\right)\left(\mathrm{FNP}\right)+2\;\mathrm{oxidants}\to \left({\mathrm{RO}}_{2}\right)\left(R^{\prime }O_{2}\right)\left(\mathrm{FNP}\right)$$

9 (RO2)(R′O2)(FNP)→(RO···OR′)3(FNP)+O2→(ROOR′)(FNP)

It should be noted that OOM oxidants in reaction ([Disp-formula eq8]) could either be hydroxyl/nitrate (OH/NO_3_) radicals or ozone, depending on the chemical identity of the OOM.

#### 3.1.4 Thermodynamic Feasibility of the Different Mechanisms

##### 3.1.4.1 Mechanism 1: Stability of (RO_2_)­(FNP)

The computed binding free energies for (RO_2_)­(FNP) complexes are shown in Figure 1a. Binding is moderately favorable (Δ*G* = −1.5 to −9.3 kcal mol^–1^), with consistently stronger stabilization for SA–DMA compared to SA–AM clusters. This indicates that the (RO_2_)­(FNP) complexes will have a lower evaporation rate and on average remain longer on the surface, enabling further chemistry. Although thermodynamically possible, this pathway is kinetically limited since RO_2_ lifetimes (0.01–100 s) are far shorter than the expected time scale for two RO_2_ collisions with the same FNP (∼1000 s), assuming an FNP concentration of 10^6^ particles cm^–3^. Moreover, it involves the collision between the same FNP and two RO_2_ which is also very rare case.

**Figure 1:**
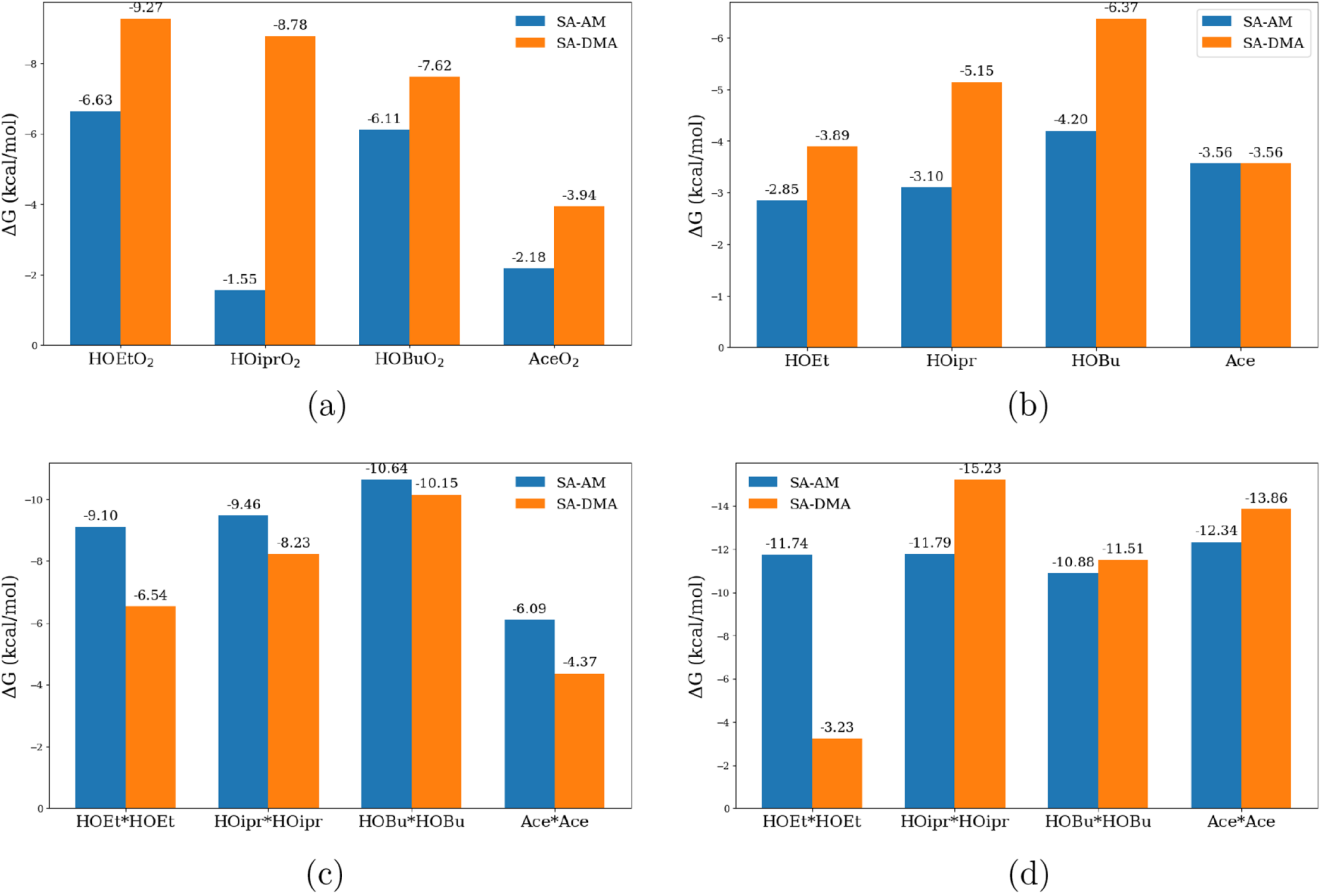
Binding energies (Δ*G*, kcal mol^–1^) of the different preceding steps before ROOR′ formation from all hypothetical mechanisms. (a) RO_2_ + FNP → (RO_2_)­(FNP); (b) OOM + FNP → (OOM)­(FNP); (c) OOM_1_ + OOM_2_ + FNP → (OOM_1_)­(OOM_2_)­(FNP); (d) RO_2_ + R′O_2_ + FNP → (RO_2_)­(R′O_2_)­(FNP).

##### 3.1.4.2 Mechanism 2: Stability of (OOM)­(FNP)

The binding of the studied OOMs to the model FNP is relatively weak, with Δ*G* values in the range of −3 to −6 kcal mol^–1^ (Figure 1b). As a result, under typical atmospheric conditions where FNP concentrations are expected to be on the order of ∼10^6^ cm^–3^ at most, the equilibrium will remain strongly shifted toward the free OOMs rather than the (OOM)­(FNP) complex. However, we note that these OOMs are relatively small model species selected for generality. Larger, highly oxidized multifunctional OOMs typically found in atmospheric aerosol growth are expected to possess stronger binding interactions, which could shift the equilibrium toward more stable complexes. Additionally, the presence of DMA in the FNP significantly enhances stabilization, consistent with its strong basicity, suggesting that DMA-stabilized particle surfaces may particularly favor initial OOM uptake and subsequent surface reactions.

##### 3.1.4.3 Mechanism 3: Stability of (OOM_1_)­(OOM_2_)­(FNP) and (RO_2_)­(R′O_2_)­(FNP)

The double OOM clusters are strongly stabilized (Δ*G* = −6 to −10.6 kcal mol^–1^). Upon oxidation, the resulting (RO_2_)­(RO_2_)­(FNP) complexes become even more favorable, reaching Δ*G* = −11.5 to −15.2 kcal mol^–1^ (shown in Figure 1c,d). While a single OOM binds more strongly to (SA)_1_(DMA)_1_ due to the higher basicity of DMA and enhanced electrostatic stabilization, simultaneous binding of two OOMs introduces steric and geometric constraints. The bulkier dimethylaminium group limits cooperative hydrogen bonding and optimal packing, whereas the more compact ammonium moiety in (SA)_1_(AM)_1_ allows greater flexibility for accommodating a second OOM, leading to stronger overall stabilization of the double-OOM complex. However, a favorable binding energy does not directly translate into an appreciable equilibrium concentration, because formation of the trimolecular cluster involves three low-concentration species:

10 $$\frac{p_{{\left(OOM\right)}_{2}FNP}}{p_{\mathrm{ref}}}={\left(\frac{p_{\mathrm{OOM}}}{p_{\mathrm{ref}}}\right)}^{2}\left(\frac{p_{\mathrm{FNP}}}{p_{\mathrm{ref}}}\right)\exp\left(-\frac{\Delta G}{RT}\right)$$

with representative atmospheric mixing ratios of FNP ≈ 10^6^ cm^–3^, OOMs in the ppb range, and RO_2_ ≈ 10^8^ cm^–3^.

Using our strongest OOM case (Δ*G* = −10.6 kcal mol^–1^), this expression yields an equilibrium concentration below 10^–4^ cm^–3^. Thus, for the OOMs considered here, essentially no (OOM)_2_(FNP) clusters would be present under atmospheric conditions, despite their thermodynamic stabilization.

This has two implications. First, while this pathway still conceptually preorganizes two OOM molecules on the FNP surface, it does not contribute under ambient conditions, because the equilibrium population of (OOM_1_)­(OOM_2_)­(FNP) clusters is surprisingly small. Second, this framework nevertheless provides a way to estimate what molecular properties would be required for mechanism 3 to become viable. Achieving even 1 cm^–3^ of (OOM)_2_(FNP) would require Δ*G* ≈ −25 kcal mol^–1^ at ppt-level OOM concentrations—approximately 15 kcal mol^–1^ stronger than the values obtained here. Assuming ∼4 kcal mol^–1^ per additional H-bonding group, this corresponds to ∼4 extra functional groups (≈2 per OOM), which is not unrealistic for highly oxygenated, multifunctional late-generation OOMs.

The oxidized RO_2_ + R′O_2_ + FNP clusters, with Δ*G* = −11 to −15 kcal mol^–1^, approach the threshold where equilibrium concentrations become non-negligible. Yet in this case, kinetics becomes the dominant limitation, because the lifetime of an adsorbed RO_2_ must be long enough for a second oxidation to occur before desorption or reaction loss. Thus, even if the thermodynamics is favorable, RO_2_ survival times pose an additional bottleneck.

In summary, Mechanism 1 is thermodynamically possible but kinetically implausible under ambient conditions. Mechanism 2 is more favorable, as OOM oxidation on FNP yields more strongly bound RO_2_ intermediates, but is still limited by desorption and radical lifetimes. Mechanism 3, in its current form, is not feasible for the simple OOMs studied here, because the equilibrium abundance of (OOM)_2_(FNP) clusters is far below 1 cm^–3^. However, the analysis highlights which molecular features would be required for mechanism 3 to become viable: significantly stronger OOM–FNP interactions (ca. −20 to −25 kcal mol^–1^) corresponding to more functionalized, higher-generation OOMs. Alternatively, a larger FNP could also lead to stronger binding of the OOM. We tested the binding of an *n*-butanol molecule to an (SA)_10_(DMA)_10_ FNP (see Supporting Information). It is found that the probability of the second OOM colliding before the first evaporates is unity. Hence, the formation via eq 7 is in fact feasible and coadsorption could still provide a plausible route to ROOR′ formation, though the need for consecutive oxidation and RO_2_ survival would remain limiting factors.

### 3.2 Structures of the ^3^(RO···OR′)­(FNP) Clusters

The triplet ^3^(RO···OR′) cluster is a short-lived intermediate formed on the FNP surface through the radical reactions discussed in the previous section. Importantly, the complex shown in eq 9 is not produced by a bimolecular clustering collision; rather, we examine the stability of the surface-bound triplet intermediate ^3^(RO···OR′)_1_(FNP)_1_ that arises after these prior reactions which is shown in eqs 3, 6 and 9.

Figure 2 shows the optimized global-minimum structures for all the ^3^(RO···OR′)­(FNP) clusters obtained from configurational sampling at the ωB97X-D/6–31++G­(d,p) level. For all the studied cluster structures we observe a proton transfer from SA to the bases.

**Figure 2:**
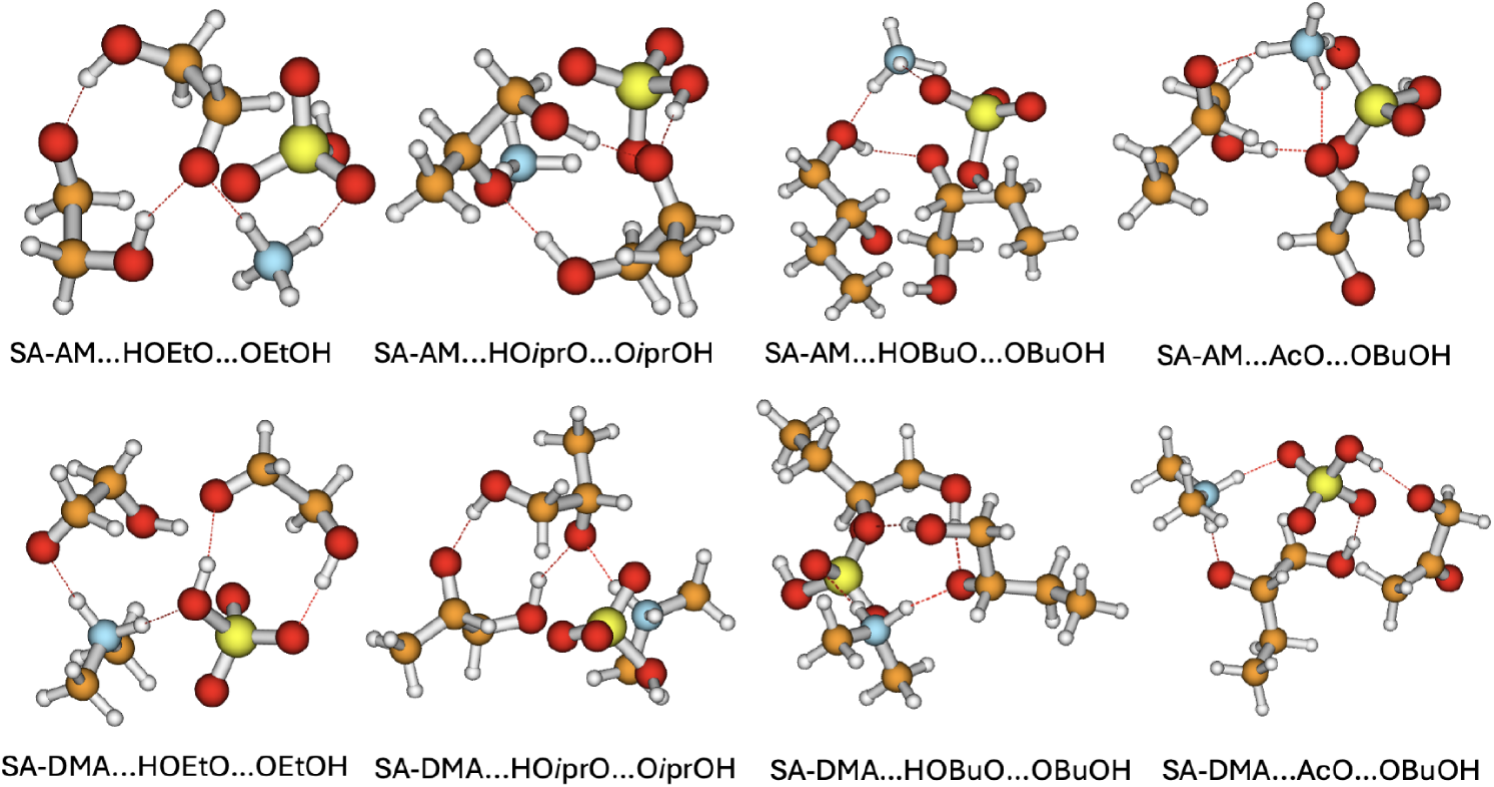
Global minima structure of the ^3^(RO···OR′)­(FNP) cluster at ωB97X-D/6–31++G­(d,p) level of theory. Color coding: Red = Oxygen, Gray = Hydrogen, Blue = Nitrogen, Yellow = Sulfur, Orange = Carbon.

The corresponding energetics for the ^3^(RO···OR′)­(FNP) clusters are given in Figure 3, illustrating how the reaction free energies change upon the binding of the organic intermediate ^3^(RO···OR′) clusters to the FNP. The key factor contributing to this increased stability is the stronger hydrogen bonding and electrostatic interactions in SA–DMA clusters. DMA, being a secondary amine with two electron-donating methyl groups, exhibits a higher proton affinity than AM. This leads to a stronger interaction with sulfuric acid, stabilizing the overall cluster structure. These factors are particularly evident in the (SA)_1_(DMA)_1_(HOBuO···OBuOH)_1_ cluster, where the binding free energy dramatically decrease from −1.87 kcal mol^–1^ for SA–AM to −10.51 kcal mol^–1^ for SA–DMA. The significant stabilization of this specific system suggests that DMA interacts more favorably with bulky organic alcohols like butanol than AM does.

**Figure 3:**
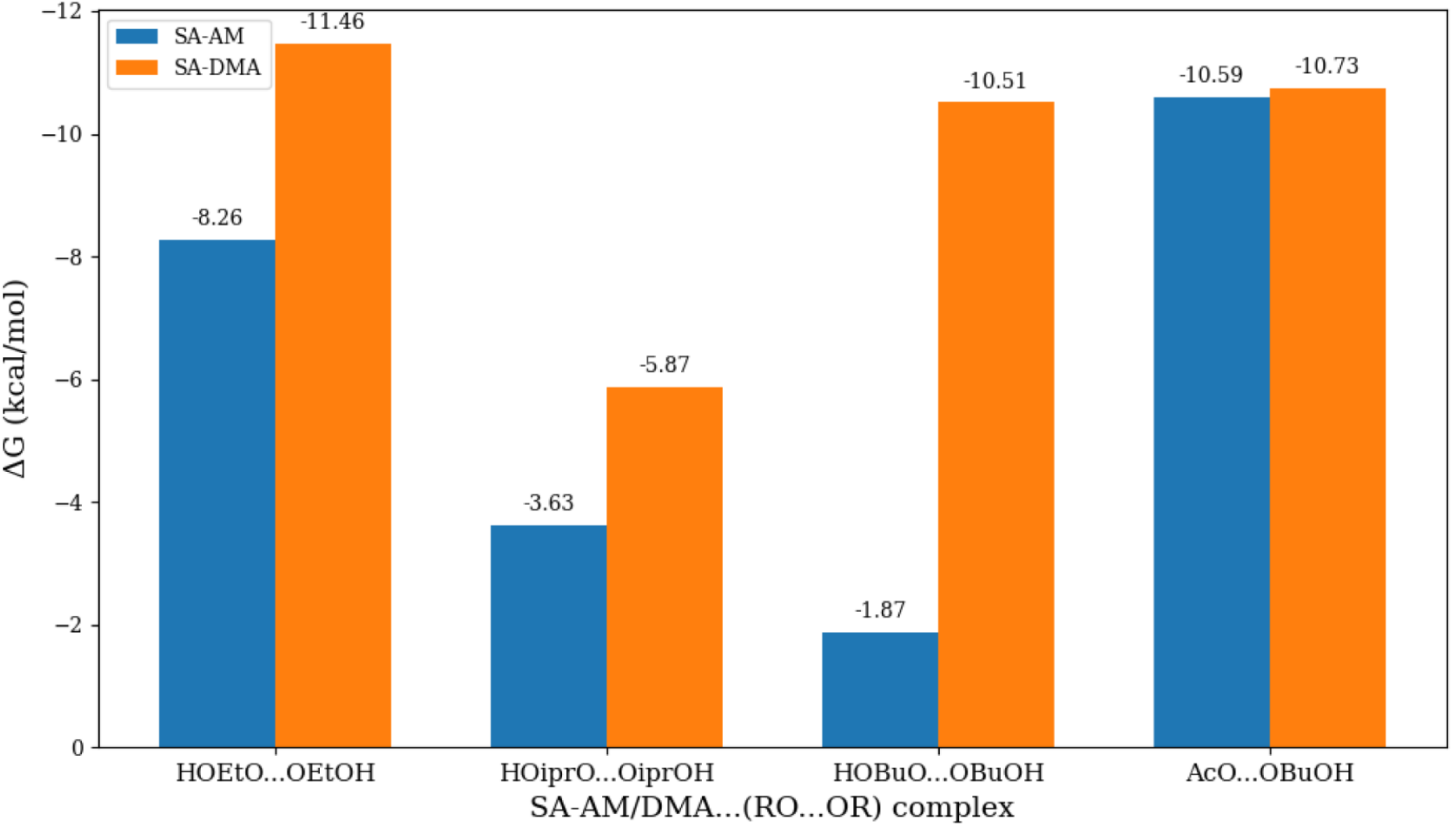
Binding free energies of the ^3^(RO···OR′)_1_(FNP) cluster relative to the ^3^(RO···OR′) + FNP calculated using the ωB97X-D/6–31++G­(d,p) level of theory.

Nevertheless, all intermediates exhibit negative binding free energies, indicating that dissociation back to ^3^(RO···OR′) + FNP is most likely limited under atmospheric conditions if the subsequent ISC rates are fast.

### 3.3 Dissociation Free Energies of the ^3^(RO···OR′)_1_(FNP)_1_ vs Isolated ^3^(RO···OR′) Clusters

From the binding free energies calculated in the previous section, the triplet clusters—if formed—are expected to exhibit reduced evaporation rates compared to isolated bimolecular complexes. However, this does not guarantee that they will inevitably evolve into extremely low-volatility ROOR′ accretion products, as alternative pathways such as alkoxy dissociation, intramolecular H-shifts, or other RO_2_-derived reactions may still compete. We next examined how the FNP model cluster further influences their stability. Specifically, we compared the free energies for dissociation of the ^3^(RO···OR′)­(FNP) clusters into RO + R′O­(FNP) with those of the isolated ^3^(RO···OR′) complexes (Figure 4). This comparison directly addresses whether the FNP environment suppresses the back-dissociation of radical fragments, thereby facilitating subsequent accretion product formation. Here, the dissociation free energy Δ*G* is defined for the reaction:

11 (RO···OR′)3(FNP)→RO+(RO′)(FNP)

**Figure 4:**
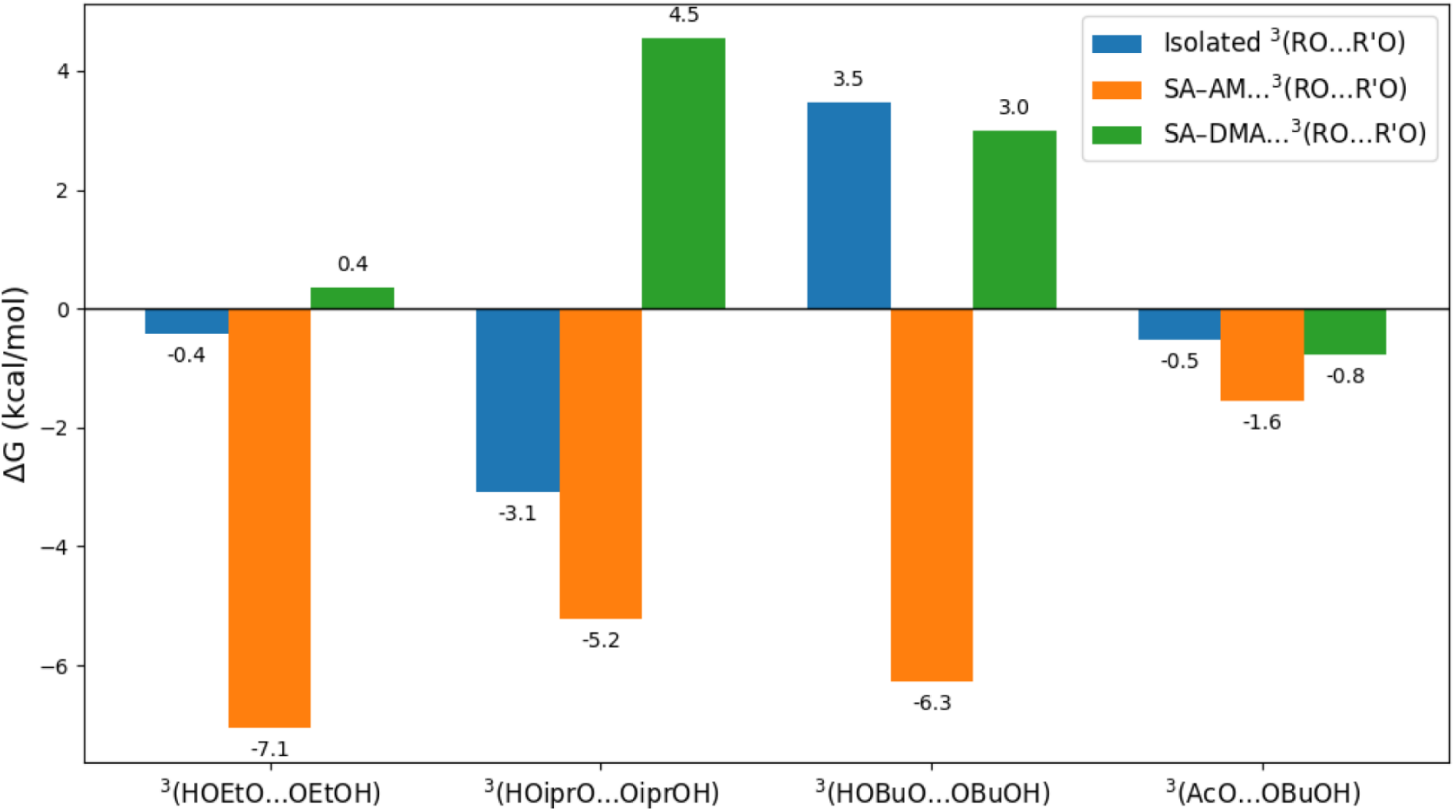
Free energies of dissociation (Δ*G*) for ^3^(RO···OR′)_1_(FNP)_1_ → (RO) + (R′O)­(FNP) (orange and green bars), compared to isolated ^3^(RO···OR′) → RO + R′O (blue bars).

This sign convention differs from that commonly used in atmospheric clustering studies, where free energies are often reported for cluster formation. Our choice reflects the fact that, under atmospheric conditions, dissociation of the alkoxy–alkoxy complex is the only physically relevant process, whereas formation via bimolecular collision of alkoxy radicals is negligible due to their low concentrations. Positive Δ*G* values thus indicate that the bound complex lies below the separated radicals in free energy, while negative values imply the opposite.

For the isolated triplet clusters, the calculated Δ*G* values are generally close to thermoneutral, and in some cases positive (e.g., HOBuO···OBuOH: + 3.5 kcal mol^–1^). This indicates that these complexes are intrinsically fragile and prone to dissociation in the absence of a stabilizing environment. By contrast, association with the FNP strongly shifts the dissociation free energies to more negative values, especially in the case of SA–AM clusters (e.g., ^3^(HOEtO···OEtOH): −7.1 kcal mol^–1^ on SA–AM vs −0.4 kcal mol^–1^ in isolation). This shows that the FNP model cluster acts as a stabilizing scaffold, effectively “gluing” the radical fragments together.

The degree of stabilization, however, depends strongly on both the organic substituents and the base molecule in the FNP cluster. Hydroxyl peroxy radicals with small, linear substituents such as ethanol are stabilized most effectively, consistent with their ability to form stronger hydrogen bonds with the acid–base cluster. Bulky substituents such as isopropyl or butyl show weaker stabilization, especially in SA–DMA clusters, where steric hindrance reduces the ability of DMA to interact optimally with the organic fragments. This is reflected in the slightly endergonic dissociation observed for ^3^(HOiprO···OiprOH) on SA–DMA (Δ*G* = +4.5 kcal mol^–1^).

The chemical nature of the base also plays a central role. While DMA is generally known to stabilize acid–base clusters more efficiently than ammonia, in the present systems its bulkiness leads to less favorable binding with certain organic radicals, in some cases even destabilizing the RO fragments. By contrast, the smaller AM can flexibly adapt to hydrogen-bonding geometries, yielding consistently exergonic dissociation free energies. These results highlight that the stabilizing effect of the FNP environment is highly sensitive to molecular structure: both the functional group identity and steric accessibility govern whether RO radicals remain bound or dissociate.

In general, for many small systems, the binding of the ^3^(RO···OR′) intermediates in the gas-phase is so weak that dissociation will be very fast (and often the dominant channel). In contrast, the association with FNP likely makes the dissociation channel uncompetitive, so ROOR′ formation now “only” competes e.g., with H-shifts. This effect is most pronounced for small, hydrogen-bonding substituents on SA–AM clusters, but less effective for bulky substituents on SA–DMA. In addition, it should be noted that the chosen −OH group as a tether is not the most strongly binding group and alkoxy radicals with either more, or stronger binding functional groups, will further suppress dissociation.

### 3.4 Kinetics of Accretion Product Formation on the FNP Model Cluster

The first step of the reaction mechanism involves the formation of a triplet ^3^(RO···OR′) cluster which subsequently crosses to the singlet surface via intersystem crossing (ISC) and recombines to form the peroxide ROOR′ accretion product. This ISC rate calculation requires the spin–orbit coupling matrix elements and the excitation energies (*E*) of the involved electronic states (*S*_1_, *S*_2_, *S*_3_, *S*_4_) from the lowest (*T*_1_) state. We calculated the accretion product formation ISC rate on the FNP model cluster and compared it to our previously reported gas-phase results.^20–23^ The overall ISC rate constants for our studied systems (corresponding to a sum of individual computed ISC rates) are given in Table 1.

**Table 1:** Inter-System Crossing Rate Constants (*k*_ISC_) for Different Organic Systems on the SA–AM Surface, SA–DMA Surface and in the Pure Gas Phase

	*k*_ISC_ (s^–1^)		
System	(SA–AM surface)	(SA–DMA surface)	(gas phase)^20–23^
HOEtO···OEtOH	5.6 × 10^7^	5.7 × 10^8^	3.6 × 10^9^
HOiprO···OiprOH	2.3 × 10^6^	4.6 × 10^9^	1.9 × 10^9^
HOBuO···OBuOH	8.9 × 10^9^	5.7 × 10^9^	2.0 × 10^9^
AceO···OBuOH	3.3 × 10^8^	3.7 × 10^6^	9.0 × 10^8^

Previous work has only been performed in the pure gas-phase^20–23^ and these studies have indicated that the ISC is extremely fast with a rate of at least 10^6^ s^–1^. The accretion product formation rates (*k*_ISC_) from different organic triplet clusters ^3^(RO···OR′) to ROOR′ exhibit significant variation between the gas phase and in the presence of (SA)­(AM) and (SA)­(DMA) clusters. Generally, we notice that the gas-phase ISC rates, calculated in our previous work, are higher (ranges 10^8^–10^9^ s^–1^)^20–23^ but the presence of an acid–base surface introduces notable variations in the rate. The presence of H-bonding functional groups tend to increase the variation of the ISC rates.

The calculated ISC rates in Table 1 shows a mixed pattern: the SA–DMA cluster exhibits faster ISC in two of the four systems, while the SA–AM cluster is faster in the other two. However, some SA–DMA systems (notably (HOEtO···OEtOH) and (HOiprO···OiprOH)) show significantly larger increases—approaching an order of magnitude—relative to their SA–AM counterparts. These exceptions suggest that DMA can substantially accelerate ISC in particular structural contexts, even though the enhancement is not universal. The acceleration of ISC by DMA in selected systems can be traced to structural effects rather than purely electronic stabilization. As in the gas phase, the overall ISC rate is mainly controlled by the *T*_1_ → *S*_1_ transition, for which the energy gap is small. Consequently, variations in ISC arise primarily from changes in the SOCME. The presence of DMA modifies the hydrogen-bonding pattern at the FNP model cluster and thereby alters the relative orientation and distance of the two radical oxygen atoms. In cases such as (HOiprO···OiprOH), this leads to improved 2*p*-orbital overlap and enhanced SOCME, whereas in systems such as (AcO···OBuOH), the binding motif imposed by DMA results in less favorable radical alignment and no acceleration of ISC.

Among the studied systems, (HOBuO···OBuOH) exhibits the highest ISC rate on the SA–AM surface (10^9^ s^–1^). In contrast, (AcO···OBuOH) shows a lower ISC rate across all environments, implying that its structural features or electronic properties do not favor efficient spin-state transitions. Notably, some gas-phase ISC rates exceed their surface-bound counterparts, indicating that confinement effects and hydrogen-bonding interactions within the FNP can either stabilize or hinder ISC depending on the specific molecular environment. This result also align with our previous gas-phase ISC rates, where we observed that ISC rates strongly depended on the different orientations of the conformers and also the stereoisomer.

The relative energies of all considered electronic states, as well as SOCME values, and ISC rates for individual transitions are presented in the Supporting Information. We find that the overall ISC rate is dominated by the ISC between *T*_1_ and *S*_1_ for all of the studied systems except for the (SA)­(AM)­(HOiprO···OiprOH) and (SA)­(AM)­(AcO···OBuOH) systems. In the former case, the SOCME between *T*_1_ and *S*_1_ is zero, due to the large distance between two radical centers^43,44^ leading to a zero ISC rate between these states and a low overall ISC rate. In the latter case, the SOCME between *T*_1_ and *S*_1_ is very small (0.45 cm^–1^) leading to slow ISC rate for this transition (*T*_1_ → *S*_1_: 2.36·10^6^ s^–1^). We also notice that there is a significantly higher energy gap between *T*_1_ and *S*_2_, *S*_3_, and *S*_4_ making transitions from these states extremely slow. These findings align with our previous work^20–23^ that when the cluster sizes increases the energy gaps between *T*_1_ and *S*_2_, *S*_3_, and *S*_4_ also become larger making the ISC rates extremely slow. However, both in previous and the present work we notice that the energy gap between *T*_1_ ans *S*_1_ are relatively low and the non negligible SOCME values between these states always lead to the high ISC rates.

### 3.5 Structures and Stabilty of the (ROOR′)­(FNP) Clusters

Finally, we calculated the reaction free energies of the singlet ROOR′ accretion products on the model FNP surface to investigate how strongly these covalently bonded dimers adhere. Hence, we look at the following reaction:

12 $${\mathrm{ROOR}}^{\prime }+\mathrm{FNP}\to \left({\mathrm{ROOR}}^{\prime }\right)\left(\mathrm{FNP}\right)$$

Configurational sampling was carried out using the approach described in Section 2.3. The optimized structures of the covalently bonded ROOR′ accretion products that interact with the FNP precursors are presented in Figure 5.

**Figure 5:**
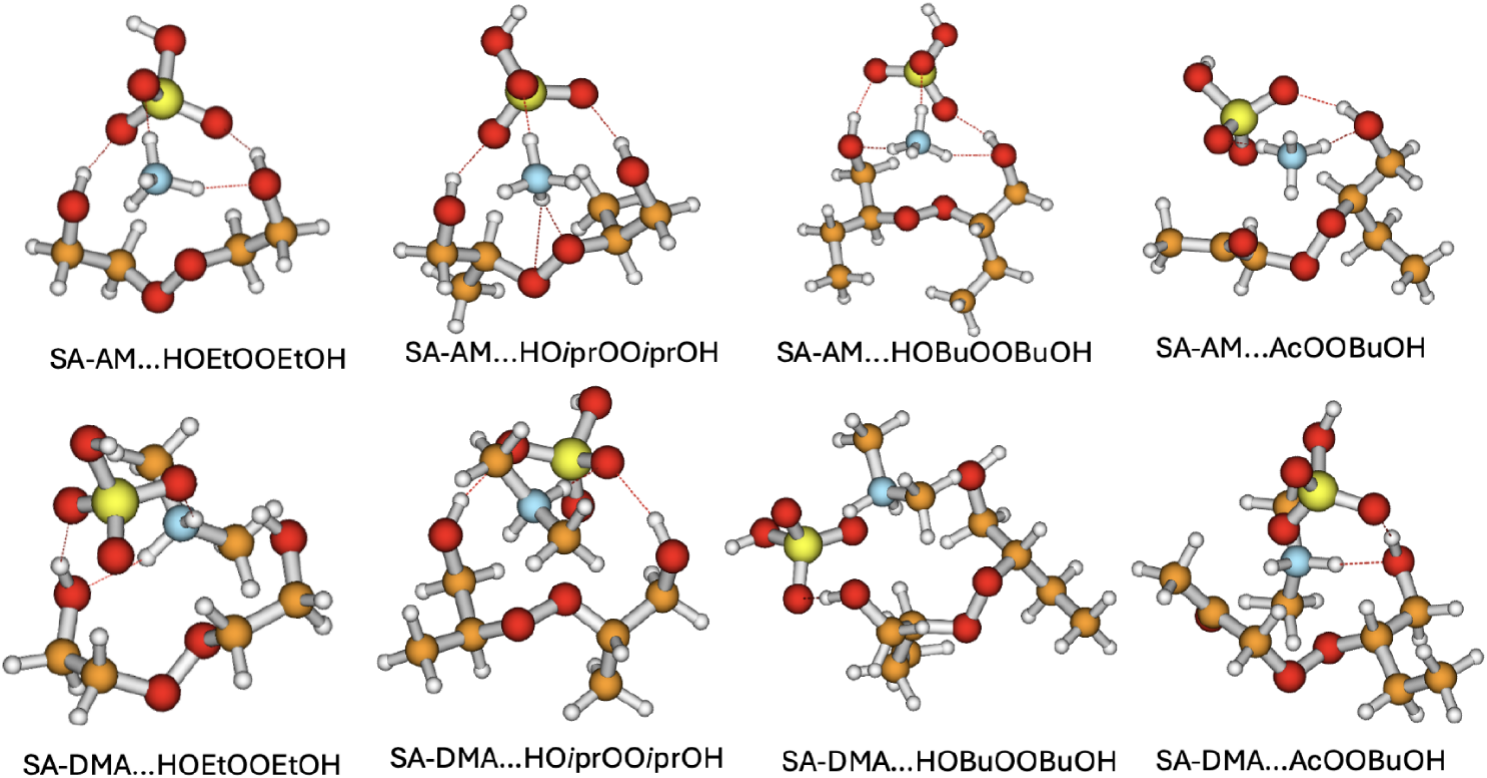
Global minima optimized structure of ROOR′-accretion–FNP cluster at ωB97X-D/6–31++G­(d,p) level of theory.

The binding free energies calculated at the ωB97X-D/6–31++G­(d,p) level of theory are shown in Figure 6. Despite DMA being a strong base, the AM-containing (ROOR′)­(FNP) clusters possess binding energies very similar to those with DMA, differing by only 1 kcal mol^–1^. This is because the ammonium ion, with its *T*_d_ symmetry, can form four intermolecular bonds—unlike DMA, which can form only two—improving coordination. Additionally, DMA’s bulky methyl groups impose steric hindrance, whereas protonated AM remains centrally embedded, forming a fully coordinated complex.

**Figure 6:**
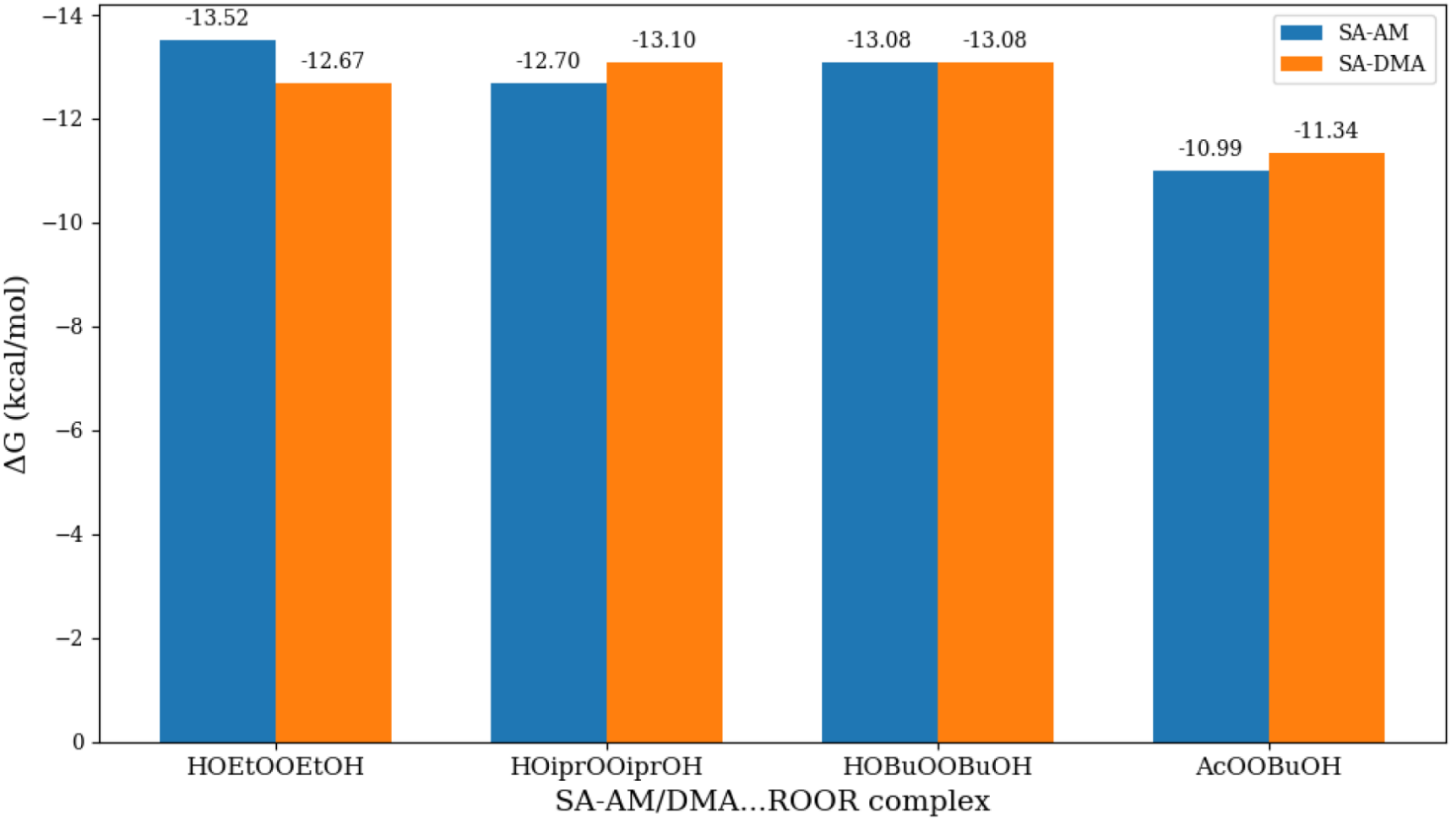
Binding free energies of the ROOR′-accretion–FNP cluster relative to ROOR′ + FNP calculated using ωB97X-D/6–31++G­(d,p) level of theory.

Across all systems, binding free energies for the (ROOR′)­(FNP) clusters range from −10.99 to −13.52 kcal mol^–1^. The data clearly shows that the (ROOR′)­(FNP) clusters exhibit significantly stronger binding to both (SA)­(AM) and (SA)­(DMA) clusters compared to their corresponding preceding steps shown in our mechanism (Scheme 1). The strong stabilization implies that once ROOR′ accretion products are formed (via whatever mechanism), they will be efficiently retained on FNP surfaces, suppressing their evaporation and enhancing particle growth. This implies that upon accretion product formation the overall cluster is stabilized. Overall this support the hypothesis that such chemical transformations in FNPs influence aerosol growth, stability, and their capacity to exchange vapors with the atmosphere. Alternatively, if the ROOR′ accretion products is formed in the gas phase and collide with an FNP, it will bind very strongly. However, we have only explored the OH group as the anchor for the clusters. RO_2_ radicals with more functional groups or containing functional groups that bind stronger will lead to more efficient ROOR′ accretion product formation and in turn stabilize the FNP.

## 4 Conclusions

We studied the ROOR′ accretion product formation through peroxy radical recombination RO_2_ + R′O_2_ reactions in the proximity of freshly nucleated particle (FNP) components. Our cost-effective systematic funneling based conformational sampling and intersystem crossing (ISC) rate calculations demonstrate that triplet ^3^(RO···OR′) intermediates are generally stabilized on sulfuric acid–base clusters, albeit with substantial variation between both different RO, and different base molecules. Subsequent conversion into covalently bonded ROOR′ accretions results in even stronger binding up to −13.5 kcal mol^–1^, indicating significant thermodynamic stabilization within the particle. Importantly, ISC rates on the FNP model cluster are similar to those in the gas phase, highlighting that surface composition and molecular structure are key factors in dictating reactivity.

We find that the FNP model cluster tends to preferentially stabilizes one of the RO fragments in the intermediate (RO···OR′) complex, thereby suppressing its dissociation back to radicals. This effect reduces the yield of the radical-forming (dissociation) channel of the RO_2_ + R′O_2_ reaction relative to other possible pathways, including but not limited to ROOR′ accretion product formation. In this way, the FNP model cluster alters the reaction branching, favoring the formation of more stable surface-bound products. Although the overall occurrence of such bimolecular RO_2_ + R′O_2_ events on FNPs remains rare, any such reaction would contribute to particle stabilization once it occurs.

This preliminary study paves the way for future research into surface-specific photochemical and bimolecular reactions under conditions relevant to the atmosphere, with particular emphasis on reactions involving oxidation products of isoprene and α-pinene.

## Supplementary Material



## Notes

The uncertainty introduced by using a simplified FNP (Section S1), the FNP accretion product formation kinetics (Section S2), and the dimer formation rate for all individual transitions (Sections S3–S5) (PDF)
