# Supplementary material for: Can Accretion Products Be Formed at the Interface of Freshly Nucleated Particles?
Source: ACS Omega. 2026 Mar 30;11(14):22099–109. doi: 10.1021/acsomega.5c13316 (PMC13084464; doi:10.1021/acsomega.5c13316)
Supplement: Supplementary file 1 [file ao5c13316_si_001.pdf]

Supporting Information
Can Accretion Products be Formed at the Interface of Freshly Nucleated Particles?

Galib Hasan,† Theo Kurtén,‡ Ivo Neefjes,† and Jonas Elm*,†

†Department of Chemistry, Aarhus University, Langelandsgade 140, 8000, Aarhus C, Denmark

‡Department of Chemistry, University of Helsinki, P.O. Box 55 (A.I. Virtanens plats 1), FIN-00014, Finland

E-mail: jelm@chem.au.dk

Phone: +45 28938085

S1 Uncertainty Introduced by using a Simplified FNP

In the present work, $(SA)_1(AM)_1$ and $(SA)_1(DMA)_1$ dimers were employed as simplified FNP models. Real atmospheric FNPs are larger and may contain additional acid–base units or other components. Increasing cluster size is expected to strengthen VOC binding due to enhanced hydrogen-bonding networks and dispersion interactions. Indeed, preliminary configurational sampling of 1-butanol on a $(SA)_{10}(DMA)_{10}$ cluster yields a binding free energy of approximately −22 kcal mol^{-1}, substantially stronger than the ≈ −6 kcal molmol^{-1} obtained for the $(SA)_1(DMA)_1$ dimer. This suggests that the present model likely underestimates surface stabilization and VOC residence times. However, the ISC rate itself, however, is primarily governed by the local electronic structure of the ^{3}(RO···OR′) cluster, particularly the $T_1 - S_1$ energy gap and the SOCME between the radical centers. While larger clusters may perturb geometries through modified hydrogen-bonding environments, the fundamental spin–orbit coupling mechanism depends mainly on the relative orientation and distance of the radical oxygen atoms. Thus, although quantitative binding energies and surface lifetimes are sensitive to cluster size, the qualitative mechanistic conclusions regarding ISC-driven ROOR′ formation are expected to remain robust.

S2 FNP Accretion Product Formation Kinetics

S2.1 System properties

We assume the following atmospheric concentrations: [OOM] = 10^{11} cm^{-3} and [FNP] = 10^6 cm^{-3}. We performed configurational sampling to find the lowest free energy structure of 1-butanol, as a stand in for a OOM, on the surface of a $[H_2SO_4]_{10}[NH(CH_3)_2]_{10}$

FNP. To ensure that the 1-butanol stays on the surface, we treated the $[H_2SO_4]_{10}[NH(CH_3)_2]_{10}$ FNP as a rigid monomer during the creation of OOM–FNP cluster configurations in ABCluster. It was confirmed visually that the final lowest free energy structure indeed has 1-butanol at the surface. The final level of theory of the configurational sampling procedure was the B97-3c method. For the lowest energy structure, the electronic energy was corrected at the LNO-CCSD(T)/aug′-cc-pVTZ level of theory. The binding free energy of the 1-butanol–$[H_2SO_4]_{10}[NH(CH_3)_2]_{10}$ cluster was found to be −22.38 kcal mol^{-1}.

Lifetimes

We calculate the lifetime of a OOM on an FNP as the inverse of the evaporation rate coefficient $\gamma[xy \to x+y]$. The evaporation rate coefficient $\gamma[xy \to x+y]$ can be obtained from the detailed balance

$$\gamma[xy \to x+y] = \beta[x+y \to xy]\frac{p_{\mathrm{ref}}}{RT}\exp\left(\frac{\Delta G^{\ominus}[xy] - \Delta G^{\ominus}[x] - \Delta G^{\ominus}[y]}{RT}\right), \tag{S1}$$

where $\beta[x+y \to xy]$ is the collision rate coefficient of the reverse process, p_{ref} is the reference pressure (taken as 101325 Pa), R is the universal gas constant, and T is the temperature (taken as 298.15 K).

The collision rate coefficient $\beta[x+y \to xy]$ in turn can be calculated from kinetic gas theory as

$$\beta_{\mathrm{HS}}(T) = \pi(R_i + R_j)^2\sqrt{\frac{8k_{\mathrm{B}}T}{\pi\mu}}, \tag{S2}$$

where $R_i + R_j$ is the sum of hard-sphere radii of the 1-butanol and FNP, and μ is their reduced mass. The radius of 1-butanol, H_2SO_4, and $NH(CH_3)_2$ can be approximated

as

$$R = \left(\frac{3m}{4\pi\rho_{\mathrm{LB}}}\right)^{1/3}, \tag{S3}$$

where ρ_{LB} is the liquid bulk density of the molecule. We assume the following liquid bulk densities:

- 1-butanol: 810 $\mathrm{kg\,m^{-3}}$
- H_2SO_4: 1830 $\mathrm{kg\,m^{-3}}$
- $NH(CH_3)_2$: 680 $\mathrm{kg\,m^{-3}}$

The radius of the $[H_2SO_4]_{10}[NH(CH_3)_2]_{10}$ cluster is finally given by

$$R = \left(\frac{3}{4\pi}\sum_{i}^{\mathrm{monomers}} V_{\mathrm{LB}}\right)^{1/3}, \tag{S4}$$

where V_{LB} is the liquid bulk volume of the H_2SO_4 and $NH(CH_3)_2$ monomers in the cluster. Here, we assume that all molecules and monomers are ideal spheres. Using the above equations, we obtain radii of 3.3 and 6.2 Å for the 1-butanol and FNP, respectively.

Plugging the estimated hard-sphere radii in Eq. (S2), we obtain $\beta_{\mathrm{HS}} = 8.49 \cdot 10^{-10}\ \mathrm{cm^3\,s^{-1}}$. Finally, plugging this, as well as $\Delta G^{\ominus}[xy] - \Delta G^{\ominus}[x] - \Delta G^{\ominus}[y] = 22.38\ \mathrm{kcal\,mol^{-1}}$, in Eq. (S1), we find $\gamma[xy \rightarrow x + y] = 1.03 \cdot 10^{-6}\ \mathrm{s^{-1}}$. The lifetime of the OOM on the cluster is then $\sim 10^6$ s, or around 11 days.

Probabilities of two OOMs on an FNP

Assuming that the collision with a second OOM and the evaporation of the first OOM are both Poisson processes, the probability of the second OOM colliding before the first

evaporates is given through the probability of competing Poisson processes:

$$P = \frac{k_{\text{col}}[\text{OOM}]}{k_{\text{col}}[\text{OOM}] + k_{\text{OOM,evap}}} \approx 1.0. \tag{S5}$$

S3 Spin-orbit Coupling: SA-AM Clusters

Table S1: The SOCME (cm^{-1}), Energy Gap (cm^{-1}), and k_{ISC} (s^{-1}) computed for SA-AM... (OHEtO... OetOH) cluster.

Transition	SOCME (cm^{-1})	Energy Gap (cm^{-1})	k_{ISC} (s^{-1})
$T_1 \to S_1$	2.3	1038.863	5.63E+07
$T_1 \to S_2$	99.66	5207.79	1.94E+02
$T_1 \to S_3$	99.3	6758.706	1.08E-01
$T_1 \to S_4$	2.7	10142.39	6.47E-12
		Total rate	5.63E+07

Table S2: The SOCME (cm^{-1}), Energy Gap (cm^{-1}), and k_{ISC} (s^{-1}) computed for SA-AM... (OHiprO... OiprOH) cluster.

Transition	SOCME (cm^{-1})	Energy Gap (cm^{-1})	k_{ISC} (s^{-1})
$T_1 \to S_1$	0.45	1020.081	2.36E+06
$T_1 \to S_2$	63.57	6258.098	4.95E-01
$T_1 \to S_3$	121.07	7466.796	5.27E-03
$T_1 \to S_4$	0.18	9636.146	3.31E-13
		Total rate	2.36E+06

Table S3: The SOCME (cm^{-1}), Energy Gap (cm^{-1}), and k_{ISC} (s^{-1}) computed for SA-AM... (AceO... OBuOH) cluster.

Transition	SOCME (cm^{-1})	Energy Gap (cm^{-1})	k_{ISC} (s^{-1})
$T_1 \to S_1$	0.45	1.93	3.21E+08
$T_1 \to S_2$	105.56	3187.628	3.72E+06
$T_1 \to S_3$	99.29	6892.872	5.65E-02
$T_1 \to S_4$	0.07	10087.727	5.66E-15
		Total rate	3.25E+08

Table S4: The SOCME (cm^{-1}), Energy Gap (cm^{-1}), and k_{ISC} (s^{-1}) computed for SA-AM…(OHEtO…OetOH) cluster.

Transition	SOCME (cm^{-1})	Energy Gap (cm^{-1})	k_{ISC} (s^{-1})
$T_1 \rightarrow S_1$	2.36	0	8.91E+09
$T_1 \rightarrow S_2$	98.62	2893.534	1.34E+07
$T_1 \rightarrow S_3$	108.81	3458.856	1.07E+06
$T_1 \rightarrow S_4$	1.3	6272.351	1.93E-04
		Total rate	8.91E+09

S4 Spin-orbit Coupling: SA-DMA Clusters

Table S5: The SOCME (cm^{-1}), Energy Gap (cm^{-1}), and k_{ISC} (s^{-1}) computed for SA-DMA…(OHEtO…OetOH) cluster.

Transition	SOCME (cm^{-1})	Energy Gap (cm^{-1})	k_{ISC} (s^{-1})
$T_1 \rightarrow S_1$	7.3	1038.863	5.67E+08
$T_1 \rightarrow S_2$	103.08	3124.37	4.81E+06
$T_1 \rightarrow S_3$	104.92	4657.71	3.05E+03
$T_1 \rightarrow S_4$	0.95	7798.16	6.55E-08
		Total rate	5.72E+08

Table S6: The SOCME (cm^{-1}), Energy Gap (cm^{-1}), and k_{ISC} (s^{-1}) computed for SA-DMA…(OHiPrO…OiPrOH) cluster.

Transition	SOCME (cm^{-1})	Energy Gap (cm^{-1})	k_{ISC} (s^{-1})
$T_1 \rightarrow S_1$	1.7	0	4.62E+09
$T_1 \rightarrow S_2$	101.21	6656.495	1.84E-01
$T_1 \rightarrow S_3$	93.3	10591.764	8.83E-10
$T_1 \rightarrow S_4$	1.52	1.76E+04	4.44E-28
		Total rate	4.62E+09

Table S7: The SOCME (cm^{-1}), Energy Gap (cm^{-1}), and k_{ISC} (s^{-1}) computed for SA-DMA. . . (AcO. . . OBuOH) cluster.

Transition	SOCME (cm^{-1})	Energy Gap (cm^{-1})	k_{ISC} (s^{-1})
$T_1 \rightarrow S_1$	0	1.93	0.00E+00
$T_1 \rightarrow S_2$	105.56	3187.628	3.72E+06
$T_1 \rightarrow S_3$	99.29	6892.872	5.65E-02
$T_1 \rightarrow S_4$	0.07	10087.727	5.66E-15
		Total rate	3.72E+06

Table S8: The SOCME (cm^{-1}), Energy Gap (cm^{-1}), and k_{ISC} (s^{-1}) computed for SA-DMA. . . (OHBuO. . . OBuOH) cluster.

Transition	SOCME (cm^{-1})	Energy Gap (cm^{-1})	k_{ISC} (s^{-1})
$T_1 \rightarrow S_1$	1.89	0	5.72E+09
$T_1 \rightarrow S_2$	101.22	5675.5	2.09E+01
$T_1 \rightarrow S_3$	94.06	3458.856	5.88E-27
$T_1 \rightarrow S_4$	1.51	6272.351	1.98E-45
		Total rate	5.72E+09

S5 Spin-orbit Coupling: Gas-phase

Table S9: The SOCME (cm^{-1}), Energy Gap (cm^{-1}), and k_{ISC} (s^{-1}) computed for (OHEtO. . . OEtOH) cluster in the gas phase.

Transition	SOCME (cm^{-1})	Energy Gap (cm^{-1})	k_{ISC} (s^{-1})
$T_1 \rightarrow S_1$	2.22	164.17	3.57E+09
$T_1 \rightarrow S_2$	59.26	3546.549	7.28E+03
$T_1 \rightarrow S_3$	134.22	3628.066	2.35E+04
$T_1 \rightarrow S_4$	1.06	7053.497	5.10E-09
		Total rate	3.57E+09

Table S10: The SOCME (cm^{-1}), Energy Gap (cm^{-1}), and k_{ISC} (s^{-1}) computed for (OHiPrO...OiPrOH) cluster in the gas phase.

Transition	SOCME (cm^{-1})	Energy Gap (cm^{-1})	k_{ISC} (s^{-1})
$T_1 \rightarrow S_1$	2.16	280.745	1.93E+09
$T_1 \rightarrow S_2$	65.14	4761.592	8.79E+00
$T_1 \rightarrow S_3$	128.71	4857.026	1.99E+01
$T_1 \rightarrow S_4$	1.02	9348.226	1.02E-14
		Total rate	1.93E+09

Table S11: The SOCME (cm^{-1}), Energy Gap (cm^{-1}), and k_{ISC} (s^{-1}) computed for (AcO...OBuOH) cluster in the gas phase.

Transition	SOCME (cm^{-1})	Energy Gap (cm^{-1})	k_{ISC} (s^{-1})
$T_1 \rightarrow S_1$	0.01	0.9	1.59E+05
$T_1 \rightarrow S_2$	105	1785	5.11E+08
$T_1 \rightarrow S_3$	105	2561	6.20E+06
$T_1 \rightarrow S_4$	0.2	4330	2.18E-19
		Total rate	5.18E+08

Table S12: The SOCME (cm^{-1}), Energy Gap (cm^{-1}), and k_{ISC} (s^{-1}) computed for (OHBuO...OBuOH) cluster in the gas phase.

Transition	SOCME (cm^{-1})	Energy Gap (cm^{-1})	k_{ISC} (s^{-1})
$T_1 \rightarrow S_1$	1.85	241	2E+09
$T_1 \rightarrow S_2$	71	4379	9E+02
$T_1 \rightarrow S_3$	126	4495	1.0E-01
$T_1 \rightarrow S_4$	0.98	8904	0
		Total rate	2E+09
